# Health Technology Access and Peer Support Among Digitally Engaged People Experiencing Homelessness: Qualitative Study

**DOI:** 10.2196/55415

**Published:** 2024-05-14

**Authors:** Nóra Radó, Sándor Békási, Zsuzsa Győrffy

**Affiliations:** 1 Institute of Behavioral Sciences Faculty of Medicine Semmelweis University Budapest Hungary; 2 Health Center Hungarian Charity Service of the Order of Malta Budapest Hungary

**Keywords:** digital health, homelessness, digital technology, internet, access, health equity

## Abstract

**Background:**

Although the effects of digital health are receiving wide scientific attention, very little is known about the characteristics of digitally engaged people experiencing homelessness, especially in Central and Eastern Europe. Our previous research revealed a considerable level of internet use in the homeless population of Budapest, Hungary, for general purposes (350/662, 52.9%) and medical purposes (229/664, 34.6%). Moreover, a digitally engaged subgroup was identified (129/662, 19.5%).

**Objective:**

The aim of this exploratory study was to map out the resources, attitudes, and behaviors of digitally engaged homeless individuals in relation to digital technology to set the basis for potential health policy interventions, which will enable better access to health services through strengthening of the digital components of the existing health care system.

**Methods:**

Between August 18, 2022, and October 27, 2022, a total of 12 in-depth semistructured interviews were conducted in 4 homeless shelters in Budapest, Hungary. Upon analysis by 3 independent evaluators, 2 interviews were excluded. The interviewees were chosen based on purposive sampling with predefined inclusion criteria. Thematic analysis of the transcripts was conducted.

**Results:**

In the thematic analysis, 4 main themes (attitude, access, usage patterns, and solutions for usage problems) emerged. Health-related technology use mostly appeared in health information–seeking behavior. Online search for prescribed medications (5 interviews), active ingredients of medications (4 interviews), medicinal herbs believed to replace certain pills (2 interviews) or foods, and natural materials (1 interview) were mentioned. Moreover, mobile health app use (3 interviews) was reported. The intention to circumvent or check on mainstream health care solutions was mainly associated with previous negative experiences in the health care system. Several gaps in the daily use of technology were identified by the interviewees; however, more than half of the interviewees (6/10) turned out to be contact points for their peers for digital problem-solving or basic digital literacy skill enhancement in the homeless shelters. Furthermore, a lack of institutional support or special programs targeting senior clients was noted.

**Conclusions:**

Digitally engaged homeless individuals might become mediators between their peers and comprehensive digital health programs. They have the trust of their peers, can recognize and harness the benefits of digital technology, and are able to provide meaningful help in technology- and usage-related issues through experience. Digital health services have great promise in community shelters for managing and preventing health issues, and digitally engaged individuals might be important for the success of such services.

## Introduction

### The Digital Health Paradox

By the end of 2022, the number of mobile service subscribers climbed to over 5.4 billion people globally, including 4.4 billion people who also used mobile internet, and the usage gap has narrowed markedly in the last 5 years (from 50% in 2017 to 41% in 2022 on average) [1]. As of October 2023, around 5.3 billion people use the internet worldwide, which is equivalent to 65.7% of the total population of the world, and in the last year, 189 million new members joined the global community of internet users [2]. These are unprecedented numbers. Digitization, especially the adoption of digital health technologies at scale, has been boosted by the COVID pandemic since 2020, promising access to health care systems and beneficial health outcomes.

However, there is a growing body of evidence indicating that greater reliance on digital tools has the potential to widen the gap between those who have digital skills and access to digital tools and those who do not, thereby increasing already existing health inequalities [3]. Although digital solutions might be designed following guidelines, such as the World Health Organization (WHO) Global Strategy on Digital Health 2020-2025, which states that “digital health should be an integral part of health priorities and benefit people in a way that is ethical, safe, secure, reliable, equitable, and sustainable,” certain groups are unintentionally left out of the digitization boom [4]. Paradoxically, these groups often represent patients with complex psychosocial needs, specific sociodemographic characteristics, and multiple chronic conditions, and they would benefit the most from the use of digital health technologies [5-8]. van Kessel et al [6] have referred to this as the digital health paradox.

### Vulnerable Groups, Homelessness, and Health Disparities

The abovementioned groups might represent vulnerable populations that are already experiencing negative health outcomes due to their detrimental social determinants of health. This has been defined by the WHO as “the forces and systems shaping the collective conditions in which people are born, grow, work, live, and age, as well as the conditions of their daily lives” [9], and they are shaped by the distributions of money, power, and other resources [10]. Emerging research shows that there is a strong relationship between socioeconomic factors, geography, demographics, and health, with poverty, housing problems, food insecurity, abuse, gender, and ethnicity creating chronic stress, which can leave the human organism with maladaptive mechanisms that result in damage to the body’s functioning systems [11,12]. These have been linked to hypertension, premature aging, cardiovascular disease, type 2 diabetes, stroke, cancer, pulmonary disease, kidney disease, and many other health problems [10,13].

In the case of people experiencing homelessness, a complex set of social determinants of health are at play, which amplify each other’s impacts and leave this vulnerable group at the extreme low end of health outcomes, health care access, and health literacy. According to previous research, living without adequate housing options is associated with significantly higher rates of bacterial and viral infections, diabetes, hypertension, cardiovascular disease, mental health issues, and problematic substance use compared to populations with adequate housing options [14-16]. The COVID-19 pandemic has also increased the vulnerabilities and health risks of people experiencing homelessness [17].

Life expectancy data for people experiencing homelessness compared to the general population also support these findings. In a systematic review, Aldridge et al [18] found that socially excluded populations have an 8 times higher mortality rate for men and 12 times higher rate for women than the average population. In Western high-income countries, studies have shown that homelessness is an independent risk factor for mortality, and life expectancy varies between 50 and 65 years on average [19].

When considering health care access, homeless populations frequently experience structural barriers to obtain health care, including lack of health insurance in countries without universal health insurance, as well as competing interests in health care settings to their disadvantage alongside their own financial difficulties and competing priorities, which might lead them to secure food and accommodation before health care [17,20]. Research has also shown mistrust of health care systems and experiences of discrimination in care settings. Poorer health literacy measured among people experiencing homelessness compared to the general population might also lead to a poor self-rated health status and less adherence to medical recommendations and prescription medicines [21].

### Digital Health and People Experiencing Homelessness

Previous research has shown that people with lower socioeconomic status are slower to adopt new technology, and the rates of smartphone and internet use among people experiencing homelessness were lower than the rates among those with similarly low socioeconomic status but more stable housing [22]. VonHoltz et al [23] found that while experiencing homelessness, study participants showed a 68% reduction in their likelihood to access the internet compared to when they were housed. However, in terms of preferences, it was found that low-income populations, including people experiencing homelessness, rely on smartphones rather than computers for internet access owing to cost considerations, portability, and storage issues [24]. Populations at risk for limited health literacy, as indicated in the case of the homeless populations above, are also at risk for having challenges with digital technology [25].

Previous research has mentioned that it would be beneficial to equip people experiencing homelessness with the necessary tools to get them involved in digital health ecosystems as the costs of inclusion are significantly lower than the costs of treatment of health conditions, and the overall benefits show significance and persistence [3].

### Digital Health and Homelessness: Research in Hungary

While the associations between people experiencing homelessness and their health status are well researched, especially in English-speaking countries, such as Canada, the United Kingdom, and the United States, a lot less is known about the access of people experiencing homelessness to digital health tools, their digital health literacy, their attitudes toward digital technologies, or their overall characteristics in different local settings, such as Hungary, and about the specific groups existing within homeless populations [26,27].

For these reasons, the Digital Health Research Group at Semmelweis University and the Hungarian Charity Service of the Order of Malta (HCSOM) have undertaken an overarching research agenda aiming to uncover the relations between digital health and homeless populations in Hungary. Digital health technologies are defined as “technologies which use computing platforms, connectivity, software, and sensors for health care and related uses” [5]. Previous research has mapped out the attitudes of people experiencing homelessness in Budapest, Hungary, toward telecare services, with the main finding that trust in the general health care system is the central issue when it comes to the decision of homeless populations about whether they have trust in telecare services as well [28]. This study served as a starting point for a pilot project assessing the viability of a telecare system for homeless populations [29].

Access to digital tools and digital health literacy were measured in another survey (n=662), where the results demonstrated that a significant proportion of people experiencing homelessness in Budapest, Hungary, were using the internet (52.9%), while the proportion was 81.3% in a representative sample of the Hungarian population that was used as a reference group [30]. Moreover, 69.6% of people experiencing homelessness reported mobile phone ownership, with 39.9% adding that their phone had a smartphone function and 34.6% mentioning that they have already used the internet for medical purposes [30]. In terms of self-rated digital health literacy, 24.5% rated themselves as experienced or very experienced regarding internet use, while 21.5% self-reported having mediocre experience [30].

Based on these access and skill-related characteristics, we were able to filter out a broadly defined digitally engaged group (n=129, 19.5%). This subgroup possessed their own digital tools, had some level of digital health literacy, and was partly using these digital tools for health-related reasons. When we analyzed the group and ran chi-square tests for gender, age, education, frequency of medical visits, prevalence of chronic illnesses, shelter type, and social services, the prevalence of chronic illnesses (*P*=.047) was found to be an associative factor in this subgroup for the likelihood of using the internet frequently for health-related reasons. However, the quantitative survey could not discern more relevant information [30].

Thus, the main aim of this study was to map out the characteristics of this specific subgroup in order to determine (1) for what purposes and (2) how the individuals in this subgroup are using digital health technologies in the framework of an exploratory qualitative analysis.

## Methods

### Checklist

Our methodology is based on the COREQ (Consolidated Criteria for Reporting Qualitative Research) checklist as well as the methodological framework of Győrffy et al [31] (Multimedia Appendix 1). For data collection, 12 semistructured interviews were conducted.

### Ethics Approval

For all interviews, written informed consent statements were obtained, and ethics approval for the study was issued by the Scientific Research Ethics Committee of the Medical Research Council of Hungary (TUKEB 133/2020 and IV/10927/2020/EKU). In terms of the analytical framework, thematic analysis was chosen.

### Recruitment

Purposive sampling was based on the following criteria: (1) presence in the social care system of the Charity Service of the Order of Malta, (2) use of the internet every second week or more frequently, (3) internet access with own smartphone, computer, or tablet or another device with a data contract, a pay-as-you-go facility, or free Wi-Fi, (4) self-rating of an average or more competent internet user, and (5) ever use of the internet for health-related reasons. The sampling criteria of this research and the filtering criteria for the broadly defined digitally engaged subgroup in our previous research matched [30]. However, the previous research involved anonymous data collection, and the present purposive sampling did not use the previous data pool as a starting point. Thus, there may or may not be an overlap between the 2 groups.

Malterud et al [32] theorized that information power can determine the ideal sample size for qualitative studies, with a sample holding more information requiring a lower number of participants. They enlisted the following 5 criteria for analyzing information power: (1) aim of the study, (2) sample specificity, (3) use of an established theory, (4) quality of dialogue, and (5) analysis strategy. In this case, the aim of the study was to assess the specific characteristics of a subgroup of people experiencing homelessness who have a digital skillset and usage pattern (see Multimedia Appendix 2 for the interview guide), thus creating a very specific sample with limited prevalence in the overall population as measured in our previous study [30]. As a result, a smaller sample size was chosen.

In the research process, 12 interviews were conducted, but in the final analysis, 10 interviews were included, which presented all the criteria of the purposive sampling specified above. Two interviews did not contain any reference to digital health usage. At this point, this might seem as a contradiction, but people experiencing homelessness may experience literacy issues, may have somewhat limited understanding due to health issues, and may have a risk of social desirability bias in relation to interview situations, which may result in self-contradictory statements, opinions, and behaviors, in line with previous methodological findings in relation to this vulnerable population [33].

### Data Collection

Interviewees were contacted by social workers or institutional assistants at 4 shelters in the social care system of HCSOM or partner institutions. These shelters either served as a night shelter (n=1) or provided accommodation on a 24/7 basis (n=3) in Budapest, Hungary.

Based on the recommendations of the social workers or institutional assistants, one-on-one semistructured interviews were conducted between August 18 and October 27, 2022.

The interview guide was developed from experiences of the previous research, the specific study aims, and a literature review. The interviews were conducted in Hungarian with a trained interviewer. The interview guide was checked on a smaller sample of the specific subgroup (n=2) and modified based on their initial feedback.

The interview guide was based on the following topics: access to and attitude toward the health care system in general, access to and attitude toward digital tools in general and usage patterns of the internet and digital tools, and access to and attitude toward digital health and usage patterns of the internet and digital tools for health-related reasons (see Multimedia Appendix 2 for the complete interview guide).

Interviews were audio recorded in person, with an average interview length of 30 minutes. All audio-recorded interviews were transcribed verbatim, and each transcript was anonymized and assigned a unique code. The interviewer checked the transcriptions for accuracy. They were not sent back to the interviewees because people experiencing homelessness struggle with literacy challenges and Thomas et al [34] argued that evidence does not support the idea that member checking increases the credibility or trustworthiness of qualitative data [34].

### Analysis

Thematic analysis as described by Braun and Clarke was chosen as an analytical and theoretical framework [35]. In coding, we followed the “theoretical” technique in an essentialist or realist method, driven by the analytic interest to report about the experiences and realities of the study participants in relation to their engagement in a digital health ecosystem. In coding, we followed the deductive technique, that is, we worked with predetermined assumptions and themes, which followed the interview guide; however, clearly characterizable subthemes emerged around the previously identified main themes. Three independent researchers (ZG, SB, and NR) read and analyzed the data and discussed their findings.

A theoretical thematic approach was used to analyze the data and identify patterns of themes based on the checklist elaborated by Braun and Clarke [35]: (1) familiarizing with the content of the data, taking notes, and making ideas for coding based on previous assumptions and following the interview guide, (2) generating initial codes manually, (3) identifying and indexing different codes across the data set manually, (4) creating relationships between the themes and subthemes, (5) defining, mapping, and naming themes, and (6) interpreting the results.

The 3 researchers discussed and developed all themes and subthemes and clarified any discrepancies during the coding. Afterwards, they laid out the final thematic map in mutual agreement. The results are supported by participants’ anonymized quotes. Interview IDs are provided for all quotes. For each interview ID, the letter indicates the first letter of the shelter where the interview was conducted (M, Miklós utca; F, Feszty; B, Budaörs; R, REVIP) and the number indicates the serial number of the interview.

**Figure 1:**
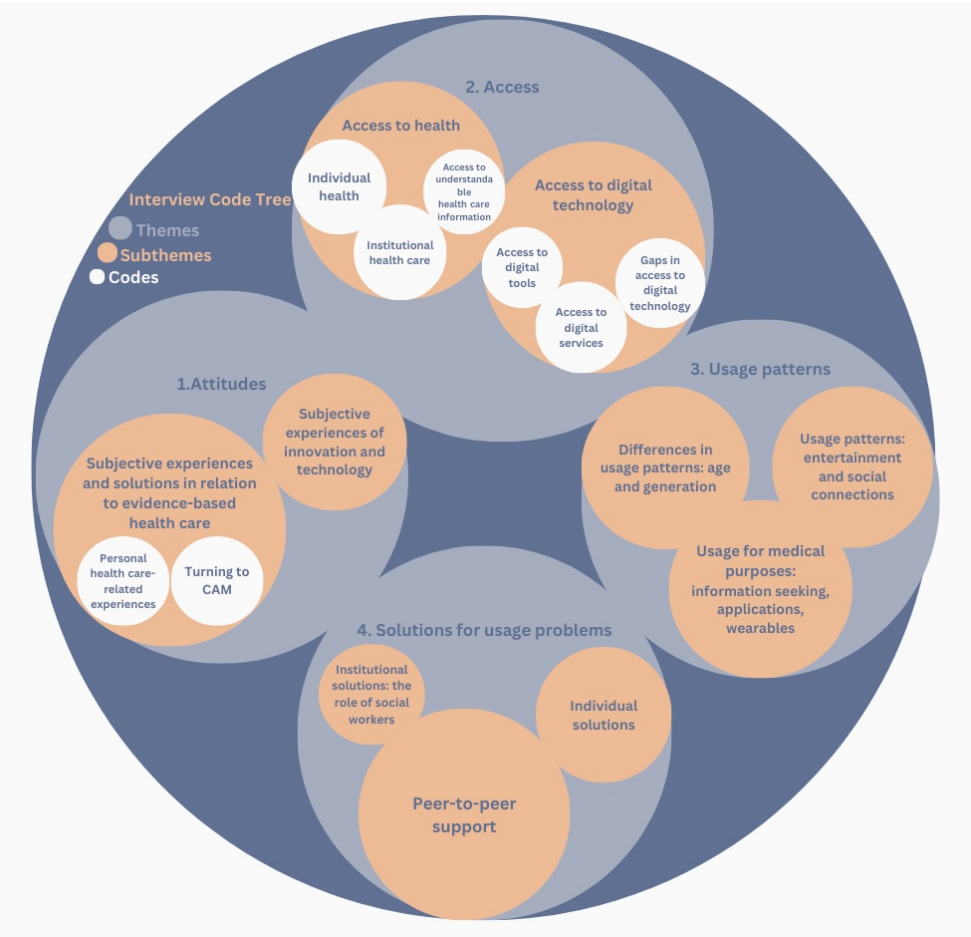
Interview code tree. CAM: complementary and alternative medical solutions.

For an overview of the themes, see Figure 1.

## Results

### Demographic Characteristics

General demographic characteristics of the sample are presented in Table 1. In terms of gender, 6 male and 4 female participants were interviewed. Older age groups were overrepresented in the sample: 1 person was <40 years old, 4 people were 40-49 years old, 2 people were 50-59 years old, and 3 people were ≥60 years old. In terms of education, high school (4 people) and vocational school (3 people) were overrepresented, while 1 person had a university education and 2 people completed primary school or below.

**Table 1:** Demographic composition of the sample.

Characteristic		Value (N=10), n (%)
**Gender**		
	Male	6 (60)
	Female	4 (40)
**Age (years)**		
	<40	1 (10)
	40-49	4 (40)
	50-59	2 (20)
	≥60	3 (30)
**Education**		
	Primary school or below	2 (20)
	Vocational school	3 (30)
	High school	4 (40)
	University	1 (10)
**Shelter**		
	HCSOM^a^ Temporary Shelter (Feszty)	2 (20)
	HCSOM Integrated Shelter (Miklós utca)	4 (40)
	Shelter House Foundation’s Night Shelter (Budaörs)	2 (20)
	REVIP Baptist Integration Center (REVIP)	2 (20)

^a^HCSOM: Hungarian Charity Service of the Order of Malta.

### Theme 1: Attitudes

#### Subtheme 1: Subjective Experiences and Solutions in Relation to Evidence-Based Health Care

##### Code 1: Personal Health Care–Related Experiences

Experiences with mainstream health care systems (hospitals, doctors, nurses, pharmacists, other medical personnel, prescription medicines, and pills) were mixed. In a minority of interviews (2/10), positive experiences with regard to access to care, quality of care, and how one was treated by the medical personnel were noted. However, the majority (7/10) reported negative experiences largely due to a negative attitude, stigmatization and mistreatment coming from the medical staff, and inadequacy of care. These signaled an overall negative attitude toward the general health care system.

I’m completely okay to be honest, I experienced that there are differences between the hospitals, I can only say that.

Interview M05

(…) …they notice where they have to go and then they have a completely different stance. Also, the emergency medical doctor, who is here, or if the ambulance services come. They behave completely differently. (…) They are condescending. Okay, we’ll do it later. Okay, come back later. And another one: do pack your stuff already, we are set to go. So, they (…) are not helpful.

Interview B09

##### Code 2: Turning to Complementary and Alternative Medicine

In a minority of interviews (3/10), turning to complementary and alternative medical solutions, medicinal herbs, or Chinese medicine was noted, which was considered as an equivalent alternative of traditional Western medicine. In parallel, in 2 interviews, a negative stance toward drugs and medicines (mentioned within the same textual context) was noted.

These shed light on the fact that interviewees sought out different potential solutions to their medical problems as some of them experienced that health care systems and traditionally produced drugs cannot and have not so far provided them with appropriate solutions. They had taken medicinal herbs or trusted ingredients, which were recommended by a trusted person or were found online.

I am aware of that, I looked up the side effects, the medicines, I will not take what they prescribe. I have already played along for long. I rather drink an herbal tea.

Interview F01

I can feel if something’s off in my body, and then I look up certain things, but to be honest, I always start with medicinal herbs, and not with pills. I go to the pharmacy, and I look up on the internet what is recommended for example for lower abdominal pain or for a story with joints.

Interview M07

#### Subtheme 2: Subjective Experiences of Innovation and Technology

Subjective experiences and attitudes toward novelties and technology were mixed. In almost half of the interviews (4/10), openness toward trying new programs and applications appeared, while in 2 interviews, a complete lack of interest was reported.

Attitudes toward the use of digital tools and the internet were also mixed. In part of the interviews, lack of trust and negative experiences were reported, for example, the risk of data misuse (1 interview), risk of making mistakes due to the autocomplete function and the speed of digital tools (1 interview), and inaccuracy of step counting (2 interviews). In another set of 4 interviews, openness toward trying new programs and applications appeared, while in a minority of interviews (2/10), lack of interest in this area was reported.

As I’m homeless at the moment, I don’t have enough (money) on my pay as you go facility that I could use the internet unlimited. Where there is free Wi-Fi, I certainly search for things I think of or what I gather from my environment, or from my godchildren. So, I want to keep up with today’s world in spite of the fact that I’m now a little bit on the brink of it.

Interview M07

You can really misuse data. I had that now, as well. Someone tapped into my bank account, abroad. I had to block access to my debit card, and I will have it done at some point.

Interview F01

As this device (tablet) works so that if my hand starts to shake just a little bit, and it gets close to it, it pulls in. And then, it writes something that I don’t want to. So, I don’t think that it is so reliable.

Interview R12

### Theme 2: Access

#### Subtheme 1: Access to Health

##### Code 1: Individual Health

The majority of interviewees (7/10) self-evaluated their health status as average or worse. Chronic diseases (cardiovascular and heart problems, and type 2 diabetes), cancerous tumors, and lasting harm from injuries were characteristic for the group. In 4 interviews, managed alcohol problems were reported. Drug abuse was not mentioned, and in 2 interviews, aversion to drugs was noted. Diagnosed mental health problems were not mentioned.

Some interviewees regularly took medicines or mentioned that their doctors prescribed them certain types of medications, which they did not take. Some interviewees made decisions in medical matters based on their own opinions and beliefs without any professional evidence.

My troubles look like heart, liver, kidney, arterial obstructions. I had deep vein thrombosis in both legs but I carried that for long. I have a very high tolerance for pain. I usually operated on myself. I froze both of my legs and I cut the ulcer out as deep as I could. Then I put herbs into the wound. It recovered within 2 weeks.

Interview F01

##### Code 2: Institutional Health Care

Interviewees were clients of 4 homeless shelters in Budapest, meaning that they had institutional access to basic health care. Their legal social security status could be provided by social institutions on the grounds of homelessness under Hungarian law. Accessible health care services included primary care (prescription and dispensing of medicines, referral to specialists, and care work), publicly funded specialized outpatient care, inpatient (hospital) care, and rescue in case of emergency.

If I have any problem, the Maltesers (HCSOM) have a doctor’s office. And if I can go there on my own feet, then I go there. If you can’t, then you will be transported to the hospital by default. There are decent people who help or call an ambulance. In the doctor’s office, they refer you to any specialist, no matter whether it’s dermatology or cardiology. Thus, they can get you to any kind of specialist.

Interview F03

##### Code 3: Access to Understandable Health Care Information

In at least one interview, a lack of access to understandable health care information was reported, and several interviewees pointed out that they were seeking out medications and ingredients online with the help of digital tools in order to understand what impacts those materials had on their body. The need for understanding health-related information was noted in at least half of the interviews in certain forms, for example, they looked up prescription medicines (5 interviews) and their ingredients (4 interviews) online, and in at least one case, they did that for their family members as well.

(….) most of the time, physicians use such Latin words in general, as lawyers do. Make it simple! No one is that much overeducated to know these. For example, laboratory tests. They should include what does this mean, sodium was X. There are some apps where you can look that up.

Interview F01

#### Subtheme 2: Access to Digital Technology

##### Code 1: Access to Digital Tools

An overwhelming majority of interviewees (7/10) used smartphones. Notebook use was reported in 1 case, and tablet use was reported in further 2 cases. One interviewee reported power bank use to charge the device.

In a minority of interviews (2/10), it was reported that in times of need, phones, tablets, and computers were sold; thus, these were not permanently accessible tools.

In this living situation, people get such digital devices much easier off their hands, if they are not in such a whacking need of them, simply to be able to make money out of it.

Interview F04

##### Code 2: Access to Digital Services

In homeless shelters, interviewees had access to the computers in possession of the shelters, and through those devices, they could get access to the internet. In certain shelters, free Wi-Fi and the option to charge their phones were available.

The majority (6/10) used free Wi-Fi inside and outside of the shelters and looked actively for options of free Wi-Fi. They could afford subscription (3 interviews) or pay-as-you-go facilities (5 interviews) less frequently. In some cases, the interviewees reported that they visited cafes in order to be able to charge their phones or use the internet (3 interviews).

(…) the Wi-Fi is so strong that you don’t have to go in and consume something, or if you go in and drink a cup of coffee or water, you get the Wi-Fi password, then sit in front of it on a bench, and it has such a strong signal that you can use it there as well, until it is open.

Interview M07

##### Code 3: Gaps in Access to Digital Technology

The interviewees reported both tool supply and network coverage as existing problems. Several interviewees mentioned the need for securing a device (smartphone) or asked about whether there was potential for decreasing the price of subscriptions and pay-as-you-go facilities. The presence of smart benches in public spaces was mentioned in 1 interview, and free Wi-Fi on trams and busses in Budapest was mentioned in another interview.

The computer park and Wi-Fi network coverage in the shelters were not mentioned as problems in the majority of interviews (7/10), and the idea of having more connectors in the building to allow easier charging surfaced in 1 interview.

Some support would be great so that a basic device could be ensured for them. And a separate health network, which is for free. For people who are ill. As there are these crisis helplines and these have green numbers.

Interview F01

… prices could be reduced (…) and for example such benches could be installed where phones can also be charged. And then you could use the Wi-Fi there.

Interview F03

It’s very difficult, I would say there could be more charging stations. The bigger shopping malls are covered, that’s fine, but what if you suddenly notice your phone is dead and you cannot go into any such places, or you are far (from the charging station), and a homeless cannot buy a ticket… How do you go there?

Interview M05

I would tell you the truth… I’m sure it would be feasible to have free Wi-Fi on busses and low-floor trams. So here, we have Wi-Fi, since this is a shelter but when we go 20 meters further, there isn’t any, the network disconnects.

Interview M05

### Theme 3: Usage Patterns

#### Subtheme 1: Differences in Usage Patterns: Age and Generations

Every interviewee used the internet at a measurable frequency on their own device. The age of the participants ranged from 35 to 69 years. The interviewer did not explicitly ask about usage characteristics by age, and the topic came up spontaneously in the case of 6 interviewees when talking about attitudes toward novelties.

In several cases, the interviewees mentioned generational differences in usage, characterizing the older generation as less involved in the digital world and less interested in novelties, while younger people were considered to be already born with digital devices, and their usage seemed to be self-evident. In 1 interview, it appeared that if there was individual motivation, then age would not pose a hinderance with regard to usage.

This is a fundamental thing, really, but many don’t know, especially the older generation. (…) So, I’m quite digital, but I’m only 40 years old for that matter. We grew up on these devices more or less already.

Interview F03

(…) I think this is age-dependent, thus generation-dependent. The elderly are okay with their basic phones. When it rings, they pick it up, then put it down. My generation already needs it more, we use it more often and the younger even more, they don’t even put it down.

Interview M05

#### Subtheme 2: Usage Patterns: Entertainment and Social Connections

Interviewees mainly used the internet for entertainment and maintaining their social relationships. Watching movies, listening to music, reading e-books, and playing phone-based games were also reported. Seven interviews mentioned Facebook and 1 interview also mentioned X (or formally Twitter) as frequently used social media sites. A minority of interviews mentioned information gathering through Wikipedia (1 interview), reading news (1 interview), and online banking (1 interview) as use cases.

I watch movies, and look up e-books, in a topic that I’m interested in. Mostly self-healing, quantum healing and such banalities.

Interview F01

I had a smartphone, so not only the music, YouTube, Facebook page is important to me, but also Wikipedia, where I can look up everything, or for example, I read a lot about various things, and the disease that I had. This is very important to me.

Interview M05

Interesting that I also keep in touch with my physician via e-mail. I had for example a CT scan, and then everything worked entirely online. I received my appointment and also the findings online. I also consider this a very positive thing, so that it is also in the cloud, and they can see it, the whole thing is much easier… I just give them my social security card (TAJ-card), and then I tell them what prescribed medication I want to have. So, I consider this absolutely positive.

Interview M05

#### Subtheme 3: Usage for Medical Purposes: Information Seeking, Applications, and Wearables

In several interviews, information seeking for medical purposes was reported. For example, interviewees looked up prescribed medications (5 interviews), active ingredients of medications (4 interviews), medicinal herbs believed to replace certain pills (2 interviews) or foods, and natural materials (1 interview). One interviewee mentioned purchasing a product believed to have medicinal value online on the basis of a Facebook advertisement.

One interviewee in their 30s communicated with the doctor about health problems via email, provided information about their illness and the prescribed medicines online, and used a health app and a step counter. These tools (health app and step counter) were also mentioned by 2 other interviewees, but one of them stopped using the step counting option as they believed it was inaccurate.

I look up the active ingredient of a pill, for example when before chemotherapy certain medicines were prescribed for me, and I looked up what kind of active ingredients they have, what side effects could they have, because a package leaflet is one thing and a real person who already had this experience and took the medicine, and what is their opinion, is another thing.

Interview M05

I already had this step counting thing, this daily fitness thing. And I remember I had a heart rate monitor in my old Samsung S5, and now I really miss that my current phone doesn’t have that anymore. (…) I also use a menstruation tracking app.

Interview M05

I usually look up online for my partner what kind of cremes and medicines there are … if they are interested what kind of ingredients the pill has, and due to his blood pressure.

Interview M06

### Theme 4: Solutions for Usage Problems

#### Subtheme 1: Individual Solutions

We included interviewees in this study who previously stated that they frequently used digital tools and self-evaluated their skills as at least average. The majority of interviewees (8/10) themselves did not mention usage problems, and when they had problems, 1 interviewee asked their family members for help but added that they preferred to solve their problems on their own.

#### Subtheme 2: Peer-to-Peer Support

It was frequently (6 interviews) reported that the interviewees offered their help to other clients who lived with them in the same shelter if they had trouble around the usage of digital tools or the internet. They solved usage-related problems for their peers, such as registration of SIM cards, activation of pay-as-you-go facilities, antivirus actions for devices, problems around online programs like Facebook and Messenger, and questions around online purchases. These user troubles represented basic problems, and the majority of interviewees (8/10) had the knowledge and skills to solve them.

Last time they wanted to buy something online, and they asked my help in that. (…) Now one of the guys from the shelter came up to me how to activate the SIM card. And then I activated it for them. Such issues are always in need.

Interview F04

There were some who asked me how to log in, how to register with an email address, how can they make a Facebook profile. Then I helped first to make an email account and then to register with that. (…) I was happy that I could help and they accepted it gladly. And then I saw that they were using it very well, they were glued onto their screens and were happy about it.

Interview M05

Usually Facebook, Messenger, or when they cannot download a game. And there is an antivirus program on every smartphone with a broom icon but they don’t know what that is. So, I tell them, pick it up and swipe with it. Clean it. And then they look at me confused. Okay, give it to me. So, then I do it, and they look. Wow, then they say, it went down to zero. Yeah, and then I say that’s the point, not to have anything on it. So there are always things like this.

Interview B09

#### Subtheme 3: Institutional Solution: Role of Social Workers

Interviewees did not report institutional solutions aiming at the development of digital skills. In 1 interview, a social worker was mentioned who provided the client with basic information on tablet use. In this case, it was the individual initiative of the social worker and not an element built into the given institution’s services.

(…) then the social worker came up to me, and taught me the basics, and then they said that I should now keep pressing the buttons around nicely, and then I’ll figure everything out by myself.

Interview R12

## Discussion

### Digital Technology and People Experiencing Homelessness

Digital technologies show a general potential for improving patient outcomes. For example, Bruce et al [36] showed that both clinical and patient-centered care outcomes were significantly better with the use of mobile health technology among 2059 orthopedic patients. However, according to a systematic catalog on digital health systematic and scoping reviews, there is less specific evidence on equitable health care (16.7%) [37].

In relation to the homeless population and digital technology, Heaslip et al [26] identified in their systemic review that mobile technology has a measurable health impact on the homeless population directly and indirectly. As an indirect impact, maintaining relations with relatives and friends as well as the outside world through entertainment, movies, and music strengthened their social connectedness and elevated their self-esteem, which in turn can have a positive impact on their personal health [38]. For the direct health impact of digital technology, they found limited evidence, with the main areas being reminders for repeat prescriptions or health care appointments. However, Heaslip et al [26] mentioned that the homeless population appears to consider that digital technology has potential health benefits, mostly in terms of online health information support and appointment reminders.

Our results partly strengthen these findings. The interviewees in our digitally engaged homeless subgroup used their digital tools primarily for entertainment purposes and to maintain their personal relationships. In terms of health care, they used their devices as new channels to reach solutions for their health problems outside the conventional health care system and to search for health-related information. However, most interestingly and most importantly, the majority of interviewees (6/10) shared that this subgroup is supporting their peers in taking up digital skills and is helping them solve their usage- and device-related problems, and this behavior has a lot of untapped potential for widening digital health usage in the homeless population.

### Health Care Needs and Personal Experiences

As indicated by the demographic characteristics, older and predominantly male interviewees shared their experiences. Consistent with the results from our previous studies [28-30], the majority of interviewees (6/10) reported multimorbidities [39,40] and having chronic diseases, such as cardiovascular diseases [41], type 2 diabetes, cancer, and permanent injuries. Older age (≥50 years) was associated with worse physical health in the homeless population, which was noted in the interviews, as the self-reported health status was regarded as average or worse [19].

In our small sample, there was no mention of mental health problems other than addictions. Previous research found that the ratio of serious mental disorders among people experiencing homelessness in Hungary was very high [42], which is in line with findings from Western countries [43]. Underdiagnosis and undertreatment of mental health problems caused by stigmatization and underperformance of the Hungarian care system might be prevalent among our interviewees as well [44]. Moreover, in line with previous studies, which estimated the prevalence of alcohol abuse at 8.5%-58.1% [45], treated alcohol problems were noted in 4 interviews; however, illicit drug use or treated drug abuse problems were not mentioned. A systematic review found that alcohol abuse is more prevalent in mainland Europe [43].

### Issues of Access to Health Care and Digital Tools

Access to primary care is resolved via the care settings of the Health Center of the HCSOM, which includes prescribing drugs, providing basic care services, and referring clients to specialists. In line with previous studies [17,20], the experiences of interviewees with accessing health care were mixed.

When looking at access to digital tools and digital services, in line with previous research, the majority of interviewees (7/10) had smartphones, which are more accessible to people with a low socioeconomic status [24]. The partial accessibility of digital devices and their use as assets in times of need as described in a minority of interviews (2/10) have been mentioned by Heaslip et al [26]. As a need, device supply was primarily mentioned by the participants, and this is in line with our previous study where 21.4% of respondents mentioned lack of a smartphone as the main barrier for not using the internet and 24.1% mentioned that availability of an appropriate device would help them use the internet more [30].

Digital services, such as computers of the shelters, were available to the participants, and in some shelters, free Wi-Fi or charging was also provided. The majority of participants (6/10) looked for free Wi-Fi options outside the shelters as well. One interviewee mentioned the lack of free Wi-Fi on public transport services and the lack of installation of smart banks in Budapest as barriers to usage. Such infrastructural problems were mentioned as causes of nonusage by 7.6% of respondents in our previous study [30]. On the other hand, several interviewees mentioned using the paid services of cafes to charge their phones or use Wi-Fi.

Several interviewees also mentioned the need for a potential decrease in internet service prices or device prices, which is in line with the finding of our previous study where 18.4% of participants said that better access to free Wi-Fi, pay-as-you-go facilities, or data contracts would help them use the internet more [30].

### Problems Around Trust

Some interviewees mentioned the feeling of being unwelcome in conventional health care settings, which is in line with previous research [41]. Some of them mentioned difficulties in getting appropriate treatment and a negative attitude from health care personnel, which might negatively influence their desire to seek health care in the future and their overall trust in the health care system, and this might explain their turn away from mainstream health care solutions.

These aspects might include a negative impact on medication adherence and an overall mistrust in mainstream medical solutions, such as taking antibiotics and chronic disease drugs, with a turn to alternative solutions. From the interviews, it was found that managing treatment themselves instead of relying on medical personnel based on their own beliefs without medical evidence was a solution. Moreover, turning to alternative and complementary medical solutions, such as homeopathy, herbal medicine, and Chinese medicine, was a way to express mistrust in conventional care settings, and digital solutions can open up a channel outside of the conventional health care system to reach such alternative solutions.

Mistrust and negative attitudes toward the health care system coupled with the need for understanding health-related language, prescription drugs, and active ingredients were associated with the main health-related use of digital tools and services in the majority of interviewees (8/10).

### Age as a Predictor for Usage and Openness

When asked about usage patterns, several interviewees spontaneously shared their views on how age differences matter in usage prevalence, outlining that older generations might be less involved and less interested in novel technologies. Several studies, including our previous quantitative research, support that age is a key sociodemographic variable that has an impact on use [29,30,46,47]. Our quantitative data showed that in access to technology, age did not seem to be a key factor; however, it might be considered as a significant factor when self-evaluating competence in digital literacy skills. This appeared in at least one of the interviews, with the respondent explaining less elevated technological skills with age.

At least three interviews indicated that age was associated with openness toward or willingness to try new technologies, which might be in line with the findings of a representative questionnaire survey (n=1500) on digital health–related knowledge, attitudes, and needs [46]. This survey was completed in 2021 and found that a quarter (26.5%) of individuals aged 65-74 years and a third (31.9%) of individuals aged older than 75 years would not like to try digital technologies in the coming years [46].

### Lack of Systematic Support Results in Peer Support for Skill-Related Problems

While interviewees recognized some support from shelters in solving infrastructural and service-related technology issues, there was a perceivable lack of systematic solutions when it came to usage-related problems and digital literacy issues. Only 1 interviewee mentioned that a social worker helped them set up their tablet and navigate through basic usage scenarios.

As we selected interviewees based on at least average self-reported digital health literacy skills and aptitude toward digital technology, with some demonstrating previous educational or professional background in IT services, their less digitally skilled peers turned to them for help.

The majority of interviewees (6/10) provided unintentional peer support in relation to technology usage issues, solved technology-related problems, and provided guidance for future scenarios. Peer support, also in this context, is defined in the literature as a process whereby individuals with lived experiences of a particular phenomenon provide support to others by explicitly drawing on their personal experiences [48]. Intentional peer support works as a formalized framework of this process that is fostered and developed by institutions, while unintentional peer support remains under the radar of institutions. The literature recognizes the potential of peer support and peer support workers, who have the necessary training and provide intentional support to their homeless peers by sharing their lived experiences in different areas of life, and members of this digitally engaged subgroup might show potential for offering peer support in digital upskilling [48,49]. Moreover, anyone considering a comprehensive digital health program for homeless groups in Hungary that concentrates on offering solutions to infrastructure and skill-related problems should take into account the untapped potential of members of digitally engaged subgroups. These individuals, through their elevated trust levels among peers, might provide better outcomes in digital upskilling than official and institutionalized digital health literacy programs. A systematic review found that empowerment and self-esteem in the homeless population increased when working with homeless peers as mentors and educators, and that peer support in general facilitates acceptance of illness and recovery and increases efficacy, social skills, and coping [50].

### Strengths

Through the qualitative analytical framework, the characteristics of a unique subgroup of digitally engaged people experiencing homelessness could be explored in a less studied area of digital health for equitable health care, where systematic mapping of review studies showed notable gaps in evidence [37].

The study aimed to enrich the still relatively small body of research concerning the characteristics, including the digital health–related characteristics, of the homeless population in Central and Eastern Europe. In North America and Western Europe, where the majority of studies involving the homeless population are conducted, the demographic composition of such populations as well as the health care system may differ significantly from Hungarian experiences, with different problems and solutions at individual and systemic levels.

### Limitations

Our study has certain limitations. As a qualitative study using in-depth semistructured interviews, the sample size was small, and this should be taken into account when drawing inferences. The study participants represented the urban homeless population from Budapest, Hungary, where socioeconomic conditions might differ from those in the countryside. The recruited homeless people had a living connection to the social infrastructure; therefore, rough sleepers and other people who were not connected to any social initiatives were not represented. The research team exclusively relied on self-reporting of digital tool access and use, and did not attempt in any way to verify these reports (eg, via phone bills, direct observation, and other methods).

In relation to people experiencing homelessness, there is an increased risk of social desirability bias when conducting interviews, meaning that respondents tend to modify their responses in the presence of an interviewer perceived to be in a different socioeconomic and overall social situation than their own [51].

### Conclusions

People experiencing homelessness can face many barriers when accessing digital technologies, including lack of appropriate devices, lack of operating infrastructure (eg, free Wi-Fi hotspots), some blind spots regarding digital skills, and a general lack of interest due to the prioritization of other basic life-supporting drives. However, in spite of all these barriers, our previous research identified a digitally engaged homeless subgroup in Budapest, Hungary, whose behaviors, usage, and access patterns were mapped in this study [30].

We found that the majority of participants (7/10) possessed a smartphone and used the often scarce pool of free Wi-Fi and the infrastructural capabilities of the shelters. Based on their articulated needs, various policy recommendations might be formulated for telephone companies and government agencies or support services. Telephone companies may consider subsidy programs to support mobile ownership and data services for this vulnerable population, as well as specific discount packages and more publicly available recharge options, as these would greatly support this group that is often in crisis and need. Government agencies may consider strengthening the infrastructural background of shelters and making free Wi-Fi accessibility an option in more public places, such as busses and piazza places, which could greatly reduce the access issues of this population. Institutional aid for accessing services and digital tools may also offer a viable option for people experiencing homelessness. A higher digital accessibility of an institution in terms of both infrastructure and digital literacy is associated with a greater likelihood of an increase in the number of digitally engaged people experiencing homelessness.

In terms of usage patterns, digitally engaged people experiencing homelessness use digital tools as an alternative information point beyond mainstream health care channels, which gives them access to check information originating from mainstream health care personnel and to seek out complementary and alternative medical solutions. These might be related to low trust in mainstream health care solutions, which might be enhanced through appropriately tailored comprehensive digital health programs. These programs could include awareness raising programs on trusted online health information sources, digital literacy and health literacy enhancing programs, and other programs to enhance their general trust in evidence-based health and the health care system.

Our most important finding is that digitally engaged homeless individuals have an aptitude for technology, and they are ready and eager to share their knowledge with their peers. This could elevate them to the role of a mediator between their peers and any potential comprehensive digital health program. Digitally engaged individuals have the trust of their peers, recognize the benefits of digital technology, and are able to provide meaningful help in technology- and usage-related issues. Thus, with appropriate training, they might become tutors for upskilling people experiencing homelessness, building a bridge between their peers and digital technologies as well as digital health ecosystems. These well-informed technologically able peers might also help enhance trust in the general health care system if their peer-to-peer support could be steered toward peer-to-peer recommendations of trusted health information sources via a specific institutional program.

Overall, our previous research showed that digital health services have great promise in community shelters for managing and preventing health issues [29,30], and this study found that digitally engaged individuals might be important for the success of such services.

## Appendix

Multimedia Appendix 1 COREQ (Consolidated Criteria for Reporting Qualitative Research) checklist.

Multimedia Appendix 2 Interview guide.

## Abbreviations

HCSOM: Hungarian Charity Service of the Order of Malta
WHO: World Health Organization
