# Supplementary material for: Health Technology Access and Peer Support Among Digitally Engaged People Experiencing Homelessness: Qualitative Study
Source: JMIR Hum Factors. 2024 May 14;11:e55415. doi: 10.2196/55415 (PMC11134250; doi:10.2196/55415)
Supplement: Multimedia Appendix 1 [file humanfactors_v11i1e55415_app1.pdf]

**Supplementary Material 1**
**Completed checklist of the consolidated criteria for reporting qualitative research (COREQ)**[1]

| No. Item | Description |
|---|---|
| **Domain 1: Research team and reflexivity** *Personal Characteristics* | |
| 1. Facilitator | Interviews were conducted by a professional interviewer (1 person) |
| 2. Credentials | MA |
| 3. Occupation | Medical Sociologist |
| 4. Gender | Female |
| 5. Experience and training | Trained interviewer; several years of experience in fieldwork |
| *Relationship with participants* | |
| 6. Relationship established | None (interviewees were unknown to the interviewers) |
| 7. Participant knowledge of the interviewer | Interviewees were informed in oral form in advance of the purpose of the interview |
| 8. Interviewer characteristics | Interests in the research topic |
| **Domain 2: study design** *Theoretical framework* | |
| 9. Methodological orientation and Theory | Theoretical thematic analysis |
| *Participant selection* | |
| 10. Sampling | Purposive sample |
| 11. Method of approach | Face-to face |
| 12. Sample size | 12 |
| 13. Nonparticipation | 0 |
| *Setting* | |
| 14. Setting of data collection | In 4 Shelters of the Charity Service of the Order of Malta |
| 15. Presence of non-participants | Only the participants and the interviewer were present |
| 16. Description of sample | Purposive sample from people experiencing homelessness, the interviews were taken between 18 August 2022 and 27 October 2022. The interviewees were chosen by social workers/ assistants working in the 4 shelters.<br><br>A total of 10 interviews were used for the analysis, 2 did not contain any reference to digital health.<br><br>The interviewees included 4 women and 6 men. In terms of age, 1 was in the youngest category, under 40, 4 in the 40-49 age group, 2 in the 50-59 age group and 3 were over 60. In terms of education, 2 interviewees completed primary school or had a lesser education, 3 completed vocational school, 4 completed high school, and 1 had a university degree. All the interviewees resided in the capital city of Hungary, Budapest. |
| *Data collection* | |
| 17. Interview guide | 2 pilot interviews were conducted. The topic guide was finalized based on the feedback, previous research, and literature review. |
| 18. Repeat interviews | N/A |
| 19. Audio/visual recording | Audio recording and transcription |
| 20. Field notes | No field notes at the setting; ex-post notes about the circumstances of the interviews |
| 21. Duration | Average interview length: 30 minutes |
| 22. Data saturation | No |
| 23. Transcripts returned | No, due to the specific interviewee sample |
| **Domain 3: analysis and findings** *Data analysis* | |
| 24. Number of data coders | 3 |
| 25. Description of the coding tree | See Figure1 |
| 26. Derivation of themes | Theoretical thematic approach |
| 27. Software | no |
| 28. Participant checking | no |
| *Reporting* | |
| 29. Quotations presented | Participant quotations are presented to illustrate the themes/findings; each quotation is identified with interview number |

| | |
|---|---|
| 30. Data and findings consistent | yes |
| 31. Clarity of major themes | yes |
| 32. Clarity of minor themes | yes |

1. *Tong A, Sainsbury P, Craig J. Consolidated criteria for reporting qualitative research (COREQ): a 32-item checklist for interviews and focus groups. International Journal for Quality in Health Care. 2007. Volume 19, Number 6: pp. 349 – 357.*
