# Supplementary material for: Health Technology Access and Peer Support Among Digitally Engaged People Experiencing Homelessness: Qualitative Study
Source: JMIR Hum Factors. 2024 May 14;11:e55415. doi: 10.2196/55415 (PMC11134250; doi:10.2196/55415)
Supplement: Multimedia Appendix 2 [file humanfactors_v11i1e55415_app2.pdf]

Supplementary Material 2 – Interview Guide

Overview of previous research:

You are now participating in a research project done by the Institute of Behavioral Sciences at Semmelweis University and the Hungarian Charity Service of the Order of Malta. The aim of the study is to map out how you use digital tools so that in the future, the healthcare service might be improved in the long run, perhaps with digital tools.

In a previous study, it turned out that many people living without stable accommodation use smartphones, apps, the internet, even for health-related purposes. So, we do this interview to get to know more about how you use such digital tools, and to collect ideas how to involve others so that they could also benefit from technology.

There is no right or wrong answer, but with every response, we will get a clearer view what we could do together for better care.

Responding to our questions is totally up to you: if you feel that you don't want to give an answer to any of the questions, that is completely okay.

Thank you for your cooperation, we appreciate your time and energy that you invested in this interview.

Questions

Demographics (Place and date of birth; Educational background; Do you consider yourself to be a homeless person at the moment?)

Health demographics (How do you consider your health in general? Do you have a chronic condition? If yes, can you talk about it? Do you take medication regularly? Have you had any problem with the healthcare system in the last year?)

Do you have a mobile phone? Do you have a smartphone? Do you share it with anyone?

Do you use the internet? If yes, where and what type of service do you use? (free Wi-Fi, pay as you go facility, subscription, other solution)

Can you regularly charge your phone? Where do you do that?

Do you remember when did you start to use your smartphone and/or the internet?

Did you get help from anyone? Did you go to any training?

What do you usually use the internet and/or your smartphone for?

Can you tell me anything that makes it difficult for your to use your smartphone and/or the internet? (not enough free Wi-Fi, not enough smartphones, no subscription or pay as you go facility, no option for charging, no computer at the shelter, cannot use online or digital tools)

Is there anything that would help you use the internet and/or your smartphone? (free Wi-Fi, charger, more smartphones, more computers, training etc.)

Do you use your smartphone and/or the internet for anything health and medicine-related?

Have you ever used a mobile health app?

Have you ever used an online registration system?

Would you use any health app, online registration system, or medical messaging system if there were one?

How do you usually reach your doctor and your nurse?

Did anything change during COVID for you? Did you have less or more health issues? Did you use your smartphone/ the internet differently?

Do you have any ideas how could the others living in the shelters be helped so that they could use the internet and/or digital tools as much as they want to?
